# Towards the Knittability of Graphene Oxide Fibres

**DOI:** 10.1038/srep14946

**Published:** 2015-10-13

**Authors:** Shayan Seyedin, Mark S. Romano, Andrew I. Minett, Joselito M. Razal

**Affiliations:** 1 Deakin University, Geelong VIC 3220, Australia, Institute for Frontier Materials; 2 Intelligent Polymer Research Institute, University of Wollongong, Wollongong, NSW 2522, Australia; 3 Department of Chemical and Biomolecular Engineering, The University of Sydney, Sydney, NSW 2000, Australia

## Abstract

Recent developments in graphene oxide fibre (GO) processing include exciting demonstrations of hand woven textile structures. However, it is uncertain whether the fibres produced can meet the processing requirements of conventional textile manufacturing. This work reports for the first time the production of highly flexible and tough GO fibres that can be knitted using textile machinery. The GO fibres are made by using a dry-jet wet-spinning method, which allows drawing of the spinning solution (the GO dispersion) in several stages of the fibre spinning process. The coagulation composition and spinning conditions are evaluated in detail, which led to the production of densely packed fibres with near-circular cross-sections and highly ordered GO domains. The results are knittable GO fibres with Young’s modulus of ~7.9 GPa, tensile strength of ~135.8 MPa, breaking strain of ~5.9%, and toughness of ~5.7 MJ m^−3^. The combination of suitable spinning method, coagulation composition, and spinning conditions led to GO fibres with remarkable toughness; the key factor in their successful knitting. This work highlights important progress in realising the full potential of GO fibres as a new class of textile.

Many exciting developments on neat graphene and graphene oxide (GO) fibres have been reported by research groups worldwide^1^,^2^,^3^,^4^,^5^,^6^,^7^,^8^,^9^. The drivers for these advancements are the ease of integration of fibres into textile research and that graphene and GO fibres provide value added properties for advanced applications. Textile research and development uses technology that has been around for many decades and is arguably one of the most feasible routes towards the development of multifunctional fabrics, including graphene-based wearable energy storage devices^10^. While there have been a few demonstrations of textile structures from GO fibres, these were woven by hand^1^,^3^,^8^,^11^. This manual production method makes it difficult to assess as to whether the process can be adopted using textile machinery and if the fibres produced are able to meet the requirements of textile processing. It is critical to study the appropriate spinning methodologies to produce knittable GO fibres and to determine the feasibility of knitting them in order to realise their full potential.

Recent developments on fibre spinning of GO have focused on improving stiffness and strength. To date, enhancements of other properties that are required to achieve knittability such as flexibility (stretchability) and toughness have been overlooked. Notably, there have been no reports on developing textile structures from GO fibres using textile machinery.

The mechanical properties of GO fibres may be tailored to suit a desired application through careful control of the fibre spinning conditions^1^,^2^,^3^,^4^,^5^,^6^,^7^,^8^,^9^. Reports on wet-spinning of liquid crystalline graphene oxide (LCGO) dispersions^1^,^12^,^13^,^14^ indicate that the alignment of LCGO domains, their interactions, as well as the structural defects inherent to the GO sheets were critical in improving the fibre properties^3^,^11^,^15^. Fibre drawing during spinning was found to result in increased alignment of LCGO domains further enhancing the mechanical properties^6^. Spinning conditions that resulted in fibre defects show low stiffness and strength^15^,^16^. In dry-jet wet-spinning of graphene nanoribbons (GNR), the introduction of an air gap between the spinneret and the coagulation bath resulted in superior mechanical properties due to the drawing of the jet (jet is herein referred to as the stable stream of spinning solution in air)^16^. This method requires that GNR is dissolved in chlorosulfonic acid and diethyl ether is used as non-solvent in the coagulation bath; both reagents pose challenges for scale-up. On the other hand, dry-jet wet-spinning of aqueous GNR dispersions resulted in fibres with inferior properties.

This work presents two significant developments: (1) how GO fibres with high flexibility and high toughness can be produced, and (2) the application of this advancement to demonstrate knitting of GO fibres into tubular textiles using a circular weft knitting machine. These developments have been achieved by careful control of various spinning parameters. We show that coagulation rate may be controlled to achieve densely packed GO fibres with near-circular cross section. Furthermore, by implementing fibre spinning strategies that enable drawing both prior to and during the coagulation process, we produced GO fibres with improved flexibility and toughness that were suitable for knitting into textile structures.

## Results

### Understanding the effects of various fibre spinning conditions on the mechanisms of fibre formation

**Figure 1:**
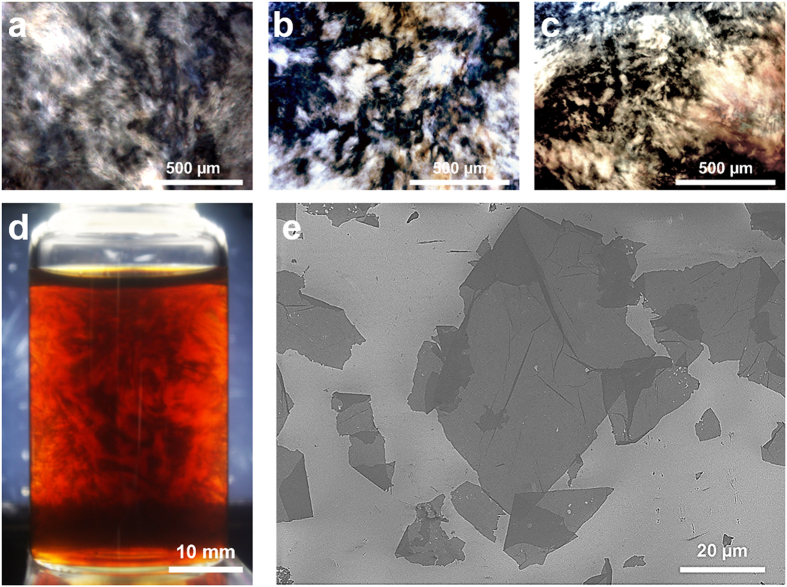
Polarised optical microscopy images showing the birefringence of LCGO spinning dispersions at various concentrations of: (**a**) ~2.8 mg mL^−1^ (as-synthesised), (**b**) 10 mg mL^−1^ and, (**c**) 20 mg mL^−1^. (**d**) The birefringence of the as-synthesised LCGO dispersion in a vial observed under cross polarised filters. (**e**) The typical SEM image of large GO sheets from as-synthesised LCGO dispersion.

The as-synthesised GO dispersion (2.8 ± 0.1 mg mL^−1^)^4^,^17^ exhibited birefringence behaviour under cross-polarisers that is typical of fully nematic liquid crystalline (LC) materials (Fig. 1a–d). GO sheets of up to 38.8 μm were measured via SEM in the as-prepared GO dispersions (Fig. 1e). The desired GO concentration for spinning (as high as 50 mg mL^−1^) was achieved through centrifugation. The GO dispersions at high concentrations also exhibited similar birefringence under cross polarisers indicating their LC properties (Fig. 1b,c). The LC behaviour of GO is the direct result of highly exfoliated and large GO sheets (*i.e.* high aspect ratio)^1^,^12^,^13^,^14^. It is this LC property that has imparted spinnability to the GO dispersions^1^,^9^.

The fibre spinning experiments involved comparing various concentrations of LCGO dispersions as spinning solutions using a range of coagulation bath formulations containing aqueous KOH solution or CaCl_2_ dissolved in either pure or mixtures of solvents such as water, ethanol, and isopropanol and evaluating their spinnability in various fibre spinning configurations.

It was generally observed that fibres spun with CaCl_2_ resulted in GO fibres with mechanical properties (*Y* = ~5.4 GPa, *σ* = ~62.9 MPa, *ε* = ~6.1%, and *T* = ~2.9 MJ m^−3^) higher than those of KOH (*Y* = ~2.9 GPa, *σ* = ~41.2 MPa, *ε* = ~3.6%, and *T* = ~1.0 MJ m^−3^). It was also noted that the mechanical properties of the GO fibres improved with CaCl_2_ concentration of up to 10 wt. % (*Y* = ~6.8 GPa, *σ* = ~108.9 MPa, *ε* = ~4.9%, and *T* = ~3.7 MJ m^−3^), which then deteriorated when increased further (see Supplementary Fig. S1). Extended soaking of the GO fibre in CaCl_2_ coagulation bath for up to 24 hrs after spinning did not result in further enhancement of mechanical properties (see Supplementary Fig. S1). These results are in agreement with previous reports^4^,^6^. The solidification mechanism in coagulation spinning is diffusion controlled^18^; thus, high CaCl_2_ concentration facilitates the diffusion of Ca^2+^ cations within the fibre. This increases the cross-linking density resulting in an enhancement of the mechanical properties. However, very high CaCl_2_ concentration in the coagulation bath can result in residual CaCl_2_ after washing and extended soaking does not induce further cross-linking since the cross-links are formed at the early stage of the coagulation.

**Figure 2:**
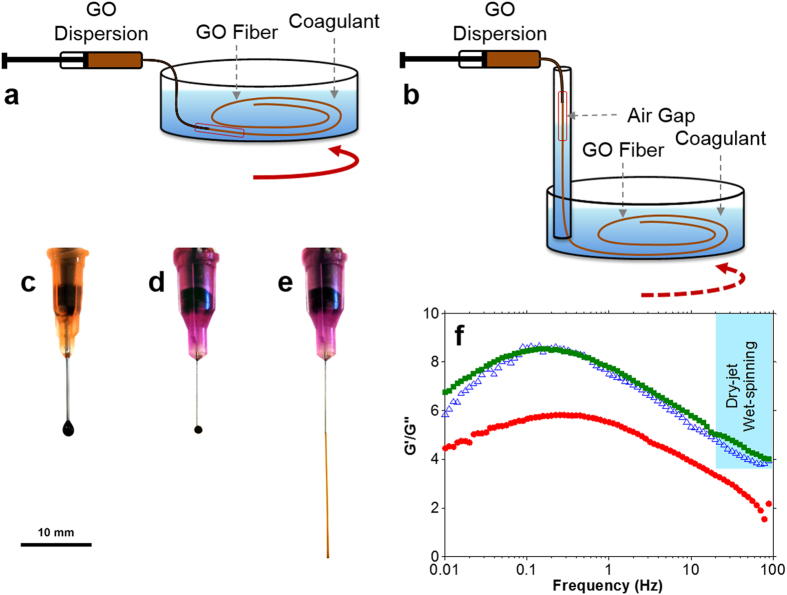
Schematic illustrations of (**a**) the wet-spinning and (**b**) the dry-jet wet-spinning methods. The coagulation bath in the dry-jet wet-spinning method can be in stationary or in rotary configurations. Droplets are formed in air when a needle with a large nozzle diameter (23 gauge) is used as the spinneret even at a high GO concentration of 20 mg mL^−1^ (**c**). A needle with a fine nozzle diameter (30 gauge) also results in droplet formation (**d**) when the GO concentration is low (5 mg mL^−1^). The continuous jet formation (**e**) for dry-jet wet-spinning is achieved using a fine needle (30 gauge) spinneret at high GO concentration (20 mg ml^−1^). (**f**) The ratio of storage modulus (*G’*) to loss modulus (*G”*) *vs*. frequency for different LCGO dispersion concentrations [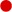 5 mg mL^−1^, 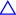 10 mg mL^−1^, and 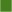 20 mg mL^−1^]. The highlighted region shows the relevant conditions in the dry-jet wet-spinning method.

The solvent composition of the coagulation bath and the fibre spinning configuration also played important roles in achieving spinnability. For example, when aqueous CaCl_2_ solution was used as the coagulation bath during wet-spinning (Fig. 2a), it was observed that the GO fibres floated in the bath. This made the fibre collection rather difficult and inhibited further processing. In the dry-jet wet-spinning configuration (Fig. 2b), the GO jet (*i.e.* the stream of GO dispersion in air) could not penetrate the coagulation bath preventing the initial fibre formation. The use of ethanol and isopropanol as CaCl_2_ solvents were also inefficient. For instance, the use of 10 wt. % CaCl_2_ in ethanol resulted in a GO fibre with irregular cross-section (see Supplementary Fig. S2) and poor mechanical properties (see Supplementary Fig. S1). When ethanol/water mixture (50/50 v/v) was used instead, the cross-section of GO fibres became regular (*i.e.* near circular) and the mechanical properties were improved (see Supplementary Fig. S1). These results imply that when choosing the non-solvent for the coagulation bath, it is crucial to consider the rate of coagulation, which is controlled by the mass transfer rate difference between solvent and non-solvent^18^,^19^. Fast coagulation rate results in irregular fibre cross-section and porous morphology, which are both associated with poor mechanical properties^3^,^15^,^16^. Since aqueous LCGO dispersions were used in this work, water is arguably the most suitable non-solvent due to the mass transfer rate difference being zero. However, it proved to be inefficient in terms of fibre processing. On the other hand, the mixture of ethanol/water (50/50 v/v) presents a low coagulation rate by which good processability, fibre circularity and dense structure formation could be attained.

It was observed that the two fibre spinning methods (wet-spinning and dry-jet wet-spinning) require different spinning conditions. In terms of spinning solution concentration, wet-spinning worked well with GO concentrations below 10 mg ml^−1^. At and above this concentration, dry-jet wet-spinning proved to be a better option in terms of ease of spinning. It is noteworthy that when the GO concentration was lower than 10 mg mL^−1^, droplets were formed at the tip of the spinning nozzle instead of a continuous jet (Fig. 2c). There are also clear differences in spinnability with spinneret nozzle diameter. In wet-spinning, GO fibres were produced using a wide range of spinneret sizes (from 34 to 21 gauge; equivalent to nozzle diameters of 0.08 to 0.51 mm, respectively). However, fibres prepared using a spinneret with nozzle diameter <0.34 mm (i.e. >23 gauge) had very small diameters (<20 μm) which were difficult to handle. In dry-jet wet-spinning, very fine spinnerets (>30 gauge, nozzle diameter <0.16 mm) were required to avoid droplet formation and to achieve spinnability (Fig. 2e). More importantly, larger nozzle diameters required higher GO concentration for the formation of a continuous jet to occur. For example, a 30 gauge needle (nozzle diameter 0.16 mm) required a 20 mg mL^−1^ GO dispersion to achieve the same spinnability as the 34 gauge needle (nozzle diameter 0.08 mm) using a 10 mg mL^−1^ GO concentration; otherwise, only droplets formed at the needle tip (Fig. 2d). Upon the investigation of nozzle size, injection rate and GO concentration during dry-jet wet-spinning, it was found that GO fibres can be successfully produced using air gap lengths of up to 5 cm. It was found however, that not all GO concentrations could be spun consistently with various nozzle sizes or at particular injection rates using a 5 cm air gap. In contrast, fibres can be consistently produced from all spinning parameters investigated when the air gap is set at 3 cm. Therefore, we maintained the air gap at 3 cm for all of our dry-jet wet-spinning experiments to ensure consistency of spinning parameters across all samples while simultaneously maximising jet-drawing. This air gap is in the range of air gaps used in commercial processes (0.5–10 cm)^20^,^21^,^22^,^23^.

The rheological properties of LCGO dispersions also determine their spinnability. In particular, the ratio of storage modulus to loss modulus (*G’*/*G”*) was previously used to describe the spinnability of LCGO dispersions at relevant frequencies^24^. These frequencies were calculated using equation (1)^25^ from the relevant shear rates (*γ*) applied during spinning. *α* is the truncation angle of the geometry (2°) during the rheological testing. Assuming the Newtonian flow of the LCGO dispersion through the spinneret, equation (2) was used to estimate the shear rate (*γ*) applied during the spinning processes, which was obtained from the injection flow rate (*Q*) and the internal radius of the needle (*r*) using equation (2)^25^.


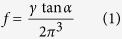



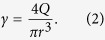


For the relevant wet-spinning conditions, i.e. using gauge 23 and 21 needles (internal radii of ~0.17 mm and ~0.26 mm, respectively) and flow rate of 10 mL h^−1^, the equivalent frequencies were calculated to be 4.1 Hz and 1.2 Hz, respectively. In contrast, dry-jet wet-spinning requires much higher frequencies of 39.1 Hz (using a gauge 30 needle and 10 mL h^−1^ flow rate) and 55.8 Hz (using a gauge 34 needle and flow rate of 2 mL h^−1^).

Shown in Fig. 2f are the frequency dependent rheological properties of three LCGO dispersion concentrations (see Supplementary Fig. S3 for each *G’* and *G”* data). For the frequencies used in the dry-jet wet-spinning (estimated to be >20 Hz based on above calculations), the 5 mg mL^−1^ LCGO dispersion exhibited a *G’*/*G”* <3 that is typical of viscoelastic soft solids^24^. In contrast, LCGO dispersions at higher concentrations (*e.g.* 10 and 20 mg mL^−1^) exhibited *G’*/*G”* >4 that is typical of viscoelastic gels^24^. These results clearly highlight the difference in rheological behaviour of GO concentrations that can be used for wet-spinning (*i.e.* below 10 mg ml^−1^) and dry-jet wet-spinning (*i.e.* above 10 mg ml^−1^) applications and how the fibre spinning conditions (*i.e.* flow rate and nozzle diameter) can be used to fine tune their flow behaviour and thus spinnability.

It is important to examine the various stages of fibre formation that can influence the final fibre property. When the LCGO dispersion passes through the spinneret, the shear applied in the capillary causes the LCGO domains to align along shear direction (referred to as shear-induced alignment)^4^,^6^,^26^. The alignment of LCGO domains can be expected to be high for small needle diameters or for high flow rates, as shown in equation (2). While shear-induced alignment of the LCGO domains is the same for the wet-spinning and dry-jet wet-spinning methods when identical spinning conditions are used, the difference in properties can arise from the differences in coagulation conditions and drawing mechanisms.

**Figure 3:**
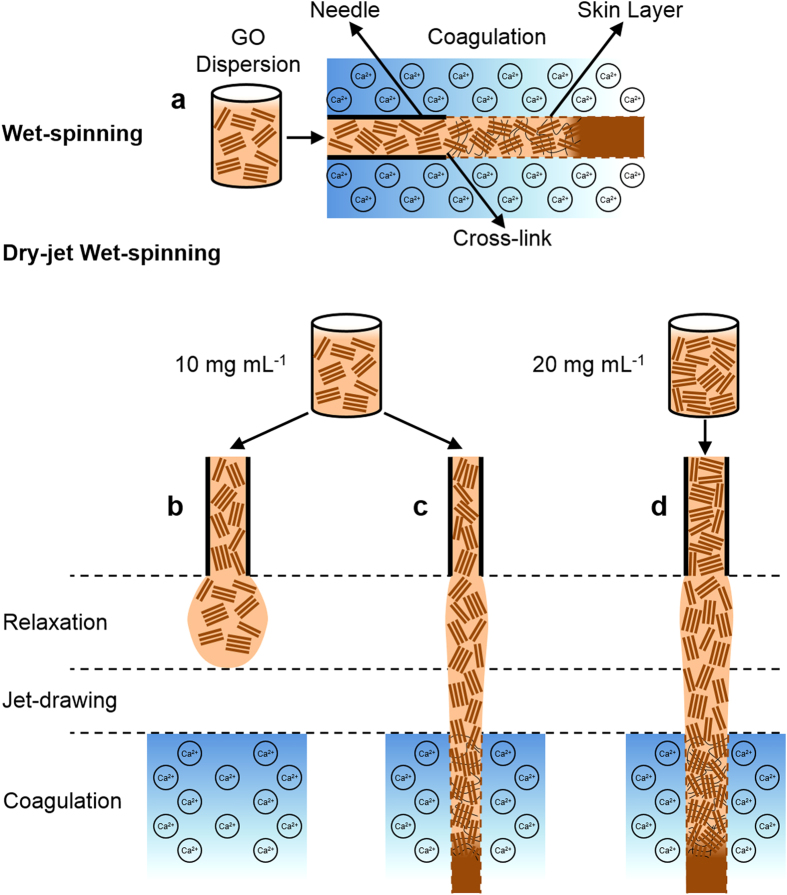
The various stages of fibre formation in wet-spinning and dry-jet wet-spinning methods are illustrated in the following schematics. (**a**) In wet-spinning, skin formation (dashed line) initially occurs at the early stage of coagulation. Further cross-linking of the internal structure (thin solid lines across GO sheets) occurs with coagulation duration. In dry-jet wet-spinning, the spinning solution can (**b**) form a droplet at the spinneret tip preventing jet formation. (**c**) Decreasing needle diameter or (**d**) increasing GO concentration can prevent droplet formation. If a continuous jet is formed in air (**c,d**), it can undergo relaxation and jet-drawing.

In wet-spinning, gelation of the jet begins immediately because the spinneret is immersed in the coagulation bath and the spinning solution is in direct contact with the coagulant as soon as it exits the nozzle tip (Fig. 2a). This gelation process may involve the formation of a “skin” around the jet (illustrated by the dashed lines in Fig. 3a) and cross-links between the GO sheets (represented by the thin solid lines across the GO sheets). As a result, the alignment of LCGO domains in the fibre is reflective of the shear-induced alignment achieved in the spinneret. Further alignment of the GO LC domains can also be achieved by gel-drawing. Gel-drawing refers to the drawing of the cross-linked gel-state fibre while in the coagulation bath. In this case, drawing is achieved through the difference between the injection rate of the spinning solution and the coagulation bath rotation speed. The efficiency of drawing in this case is limited because of the presence of cross-links.

In dry-jet wet-spinning, which uses an air gap between the spinneret and the coagulation bath, relaxation of the spinning solution can occur at the tip of the spinneret during which the alignment of the LCGO domains achieved in the spinneret may be partially lost if the die swell effect dominates. Said effect is the swelling of the spinning solution jet upon its extrusion from the spinneret, which can occur as a result of relaxation of the polymeric chains (in this case, the LCGO domains)^18^. When the GO concentration is low (<10 mg ml^−1^), the loss of alignment of the LCGO domains results in the formation of droplets (Fig. 3b). This is also known as capillary break-up^18^. We find that this effect is greatly mitigated by decreasing the nozzle diameter of the spinneret (Fig. 3c) or by increasing the GO concentration (Fig. 3d). Under these conditions, the loss of alignment of LCGO domains is quickly overcome by drawing the jet in the air gap under gravitational force (jet-drawing). The drawn GO jet then enters the coagulation bath where gelation occurs by the formation of cross-links and skin layer akin to wet-spinning. Gel-drawing may also be used in dry-jet wet-spinning. Gel-drawing *via* the rotary bath configuration was employed, as in Fig. 2b, to ensure fair comparison of experimental conditions with wet-spinning experiments.

### Fibre morphology, diameter, mechanical properties and knittability

**Figure 4:**
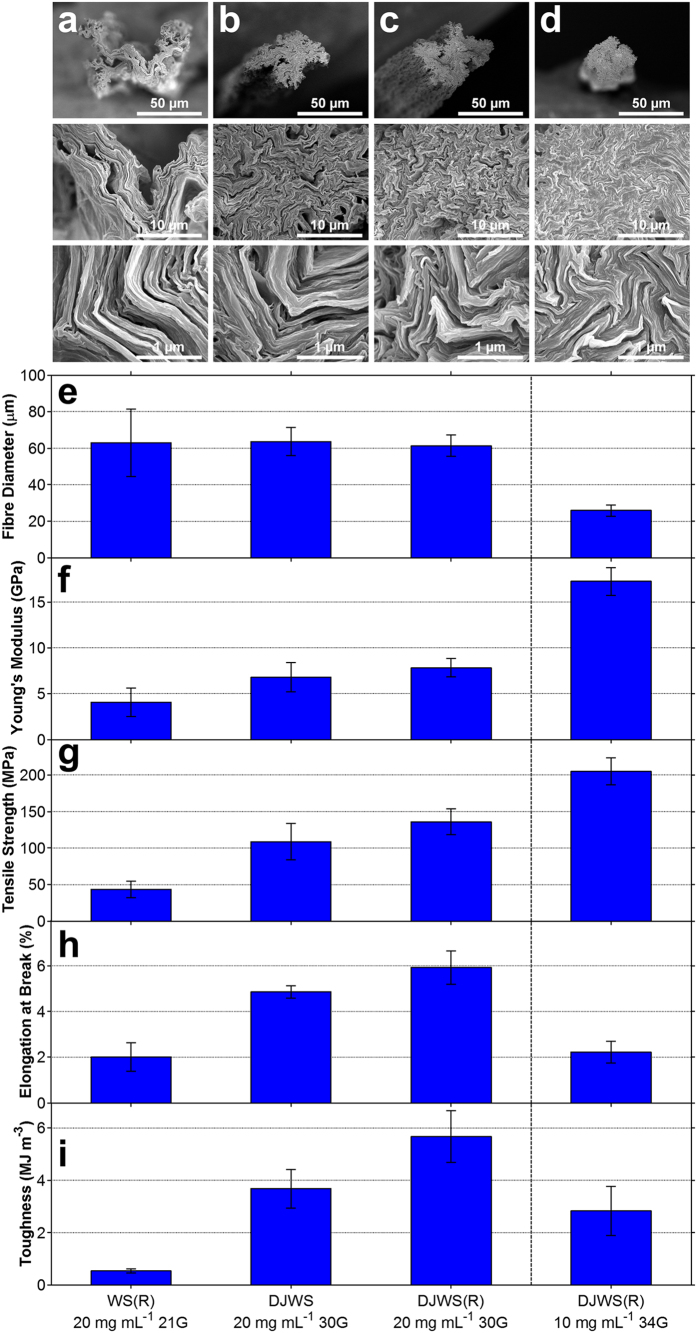
The influence of wet-spinning (WS) and dry-jet wet-spinning (DJWS) methods, spinning solution concentration, and spinneret diameter on the mechanical and morphological properties of GO fibres. Shown are: (**a–d**) cross-section morphologies of the GO fibres at various magnifications, (**e**) fibre diameter, (**f**) Young’s modulus, (**g**) tensile strength, (**h**) breaking strain, and (**i**) toughness of various GO fibres produced at different conditions. (R) in the x-axis label denotes when a rotary coagulation bath is used.

The morphologies and mechanical properties of the fibres produced by wet-spinning and dry-jet wet-spinning methods were compared by preparing GO fibres of similar diameter. This was achieved by keeping the GO dispersion concentration constant (at 20 mg mL^−1^) and only varying the spinneret size (*i.e.* using different needle gauges). It was observed that the wet-spun GO fibres exhibited irregular cross-sections and were highly porous (Fig. 4a). Fibres produced by dry-jet wet-spinning had better circularity and structural packing (*i.e.* had less voids, Fig. 4b). Furthermore, the dry-jet wet-spun fibres had superior mechanical properties than those produced by wet-spinning (Fig. 4 and Supplementary Fig. S4). For example, the dry-jet wet-spun GO fibre (fibre diameter ~63 μm) exhibited *Y* = ~6.8 GPa, *σ* = ~108.9 MPa, *ε* = ~4.9%, and *T* = ~3.7 MJ m^−3^ (Fig. 4) which were higher than the corresponding mechanical properties of the wet-spun GO fibre (*Y* = ~4.1 GPa, *σ* = ~43.5 MPa, *ε* = ~2.0%, and *T* = ~0.5 MJ m^−3^). Similar trends were observed when GO fibres with ~45 μm diameter were compared (see Supplementary Fig. S4) further confirming the superiority of dry-jet wet-spinning over wet-spinning. Supplementary Fig. S5 shows the representative uniaxial tensile stress-strain curves of the GO fibres produced at different conditions.

The observed regularity in fibre morphology (Fig. 4a) and enhanced Young’s modulus (Fig. 4f) and tensile strength (Fig. 4g) of dry-jet wet-spun fibres may be attributed to the improved alignment of the LCGO domains (due to jet-drawing) prior to cross-linking. Furthermore, the absence of jet-drawing in wet-spinning is likely to result in less alignment of the LCGO domains, which can result in less effective cross-linking and decreased interactions between the GO sheets. In contrast, the dry-jet wet-spinning method allows the formation of cross-links to the already-stretched GO jet as it enters the coagulation bath. This means that if the fibre diameter is sufficiently large to accommodate alignment of LCGO domains, cross-linking, and inter-sheet interactions, the GO fibre can demonstrate not only high stiffness and strength but also high breaking strain due to the number of sheets that can slide before the fibre breaks.

As mentioned previously, the spinning solution concentration is an important parameter that influences the spinnability of LCGO dispersions using the dry-jet wet-spinning method. Therefore, the mechanical properties of the dry-jet wet-spun fibres prepared from various GO concentrations were evaluated. It was observed that the Young’s modulus and tensile strength decreased with increasing GO concentration. The reverse was true for breaking strain; fibres prepared from high concentration spinning dispersions had higher breaking strains (see Supplementary Fig. S6). For example, the GO fibre prepared from 20 mg mL^−1^ GO dispersion exhibited *Y* = ~6.8 GPa and *σ* = ~108.9 MPa. These values are 30% and 40%, respectively, higher than that of the GO fibre spun using 50 mg mL^−1^ spinning solution. In contrast, the breaking strain of the fibre spun from 20 mg mL^−1^ (*ε* ~ 4.9%) was ~10% lower than those spun from 50 mg mL^−1^ (*ε* ~ 5.5%).

Also, the nozzle size plays a key role in determining the properties of the GO fibres. The GO fibre produced using the 34 gauge needle spinneret exhibited a higher Young’s modulus and tensile strength but lower breaking strain and toughness (*Y* ~ 11.6 GPa, *σ* ~ 133.3 MPa, *ε* ~ 2.0%, and *T* ~ 1.4 MJ m^−3^) than the GO fibre obtained from the larger 30 gauge spinneret (*Y* ~ 6.8 GPa, *σ* ~ 108.9 MPa, *ε* ~ 4.9%, and *T* ~ 3.7 MJ m^−3^) using the same GO concentration of 20 mg mL^−1^ (see Supplementary Fig. S6).

**Figure 5:**
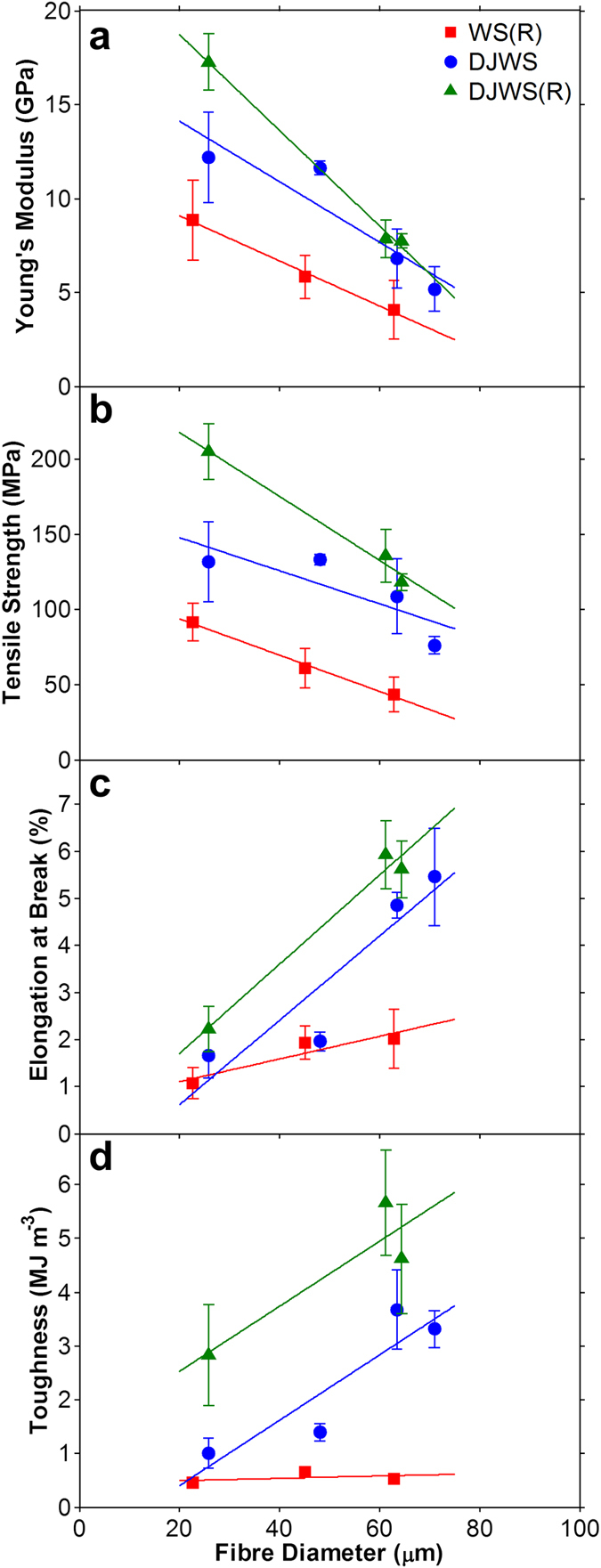
The effect of fibre diameter on (**a**) Young’s modulus, (**b**) tensile strength, (**c**) breaking strain, and (**d**) toughness of GO fibres produced by wet-spinning (WS) and dry-jet wet-spinning (DJWS) methods. (R) in the legend denotes when a rotary coagulation bath is used.

The GO fibres produced at various concentrations and spinnerets have different diameters (see Supplementary Fig. S6). Fibres with small diameters (produced by using either low GO concentration or small spinneret’s nozzle size) have high Young’s modulus and tensile strength (Fig. 5a,b) because of the decreased structural defects along the fibre length^15^,^16^ brought about by the increased alignment of the LCGO domains, packing and structural order. As the diameter of the GO fibre decreases, so does the number of GO sheets and ordered LCGO domains, hence, the defects per fibre length becomes less. Consequently, the cross-links and the van der Waals interactions between the GO sheets and ordered GO domains also becomes lower, resulting in lower slippage and therefore, decreased breaking strain and toughness (Fig. 5c,d).

Gel-drawing (*i.e.* drawing the cross-linked gel state of the fibre) employed in wet-spinning may also be used in dry-jet wet-spinning in conjunction with jet-drawing (*i.e.* drawing the jet or the uncross-linked wet-state of the fibre). To achieve a comparable gel-drawing for dry-jet wet-spinning experiments, the same rotary bath used in wet-spinning was placed at the end of the vertical tube (both filled with the coagulant) employing the same rotational speed and distance from the centre of the bath (Fig. 2b). A representative video clip of the dry-jet wet-spinning process with a rotary bath configuration is provided in the Supplementary Movie S1. Using this set-up, 20 mg mL^−1^ GO concentration and a 30 gauge needle spinneret, the fibres displayed superior mechanical properties (*Y* ~ 7.9 GPa, *σ* ~ 135.8 MPa, *ε* ~ 5.9%, and *T* ~ 5.7 MJ m^−3^, Fig. 4) compared to the fibres prepared by wet-spinning and dry-jet wet-spinning without gel-drawing. Notably, when a lower GO concentration of 10 mg mL^−1^ was spun using the finer 34 gauge needle, the fibre exhibited a circular cross-section, well-packed morphology (Fig. 4d) and remarkable stiffness (*Y* ~ 17.3 GPa) and strength (*σ* ~ 204.9 MPa). The breaking strain and toughness were, however, lower because of the smaller diameter, as discussed above. These results suggest that the mechanical properties of the GO fibres can be enhanced by combining both of the drawing mechanisms (jet-drawing and gel-drawing). Whilst GO sheet size and dispersion concentration used in this work differ from previous reports on GO fibre spinning, the mechanical properties of the dry-jet wet-spun fibres reported here (*Y* ~ 7.9 GPa, *σ* ~ 135.8 MPa, *ε* ~ 5.9%) compare reasonably well with wet-spun GO fibres in other reports (*Y* ~ 6.3 GPa, *σ* ~ 364.4 MPa, and *ε* ~ 6.8%)^6^. The higher stiffness of GO fibres produced by dry-jet wet-spinning may be attributed to the enhanced alignment of the LCGO domains during spinning. The reason for the differences in *σ* and *ε* can be due to the higher water content in coagulation bath in the previous work, which enabled production of GO fibre with near circular cross section.

Figure 5 compares how the mechanical properties change with fibre diameter for the three spinning methods. It can be seen that the rate of diameter change (*i.e.* the slope of each trend line) varies with the spinning method used, which further confirms that varying degrees of alignment of LCGO domains, fibre regularity, and structural packing of GO sheets are attained for each spinning condition and fibre diameter. The results show that the combined jet-drawing and gel-drawing (only possible through the dry-jet wet-spinning with rotary bath) yielded the highest Young’s modulus and tensile strength fibres at all fibre diameters. At any given fibre diameter, it is also shown that jet-drawing alone (*via* dry-jet wet-spinning) is consistently better than gel-drawing alone (*via* wet-spinning). Cross-linking at the more aligned state of the LCGO domains has also provided the dry-jet wet-spinning method with rotary bath a significantly higher breaking strain and thus higher toughness at each fibre diameter compared to the other spinning methods. It is noted here that the fibre diameter analyses were based on optical microscopy measurements. It is likely for the diameter measurement to have occurred along the wider dimension of an irregular fibre. These biased measurements towards the wider dimension can result in an overestimation of the fibre diameter measurement and underestimation of the Young’s modulus and tensile strength.

To demonstrate the advantages of achieving control over the GO fibre properties, selected fibres were used for knitting into textiles. Knitting was chosen because it is a simple and highly productive method of forming fabrics. It does not require special yarn packaging (*e.g.* spools) and has relatively low production time. In contrast to weaving, in which the strength of the fibre/yarn is important, knitting requires that the fibre/yarn exhibits high breaking strain and high toughness^27^. Knitting also requires the absence of defects or unevenness along the fibre/yarn length^27^.

**Figure 6:**
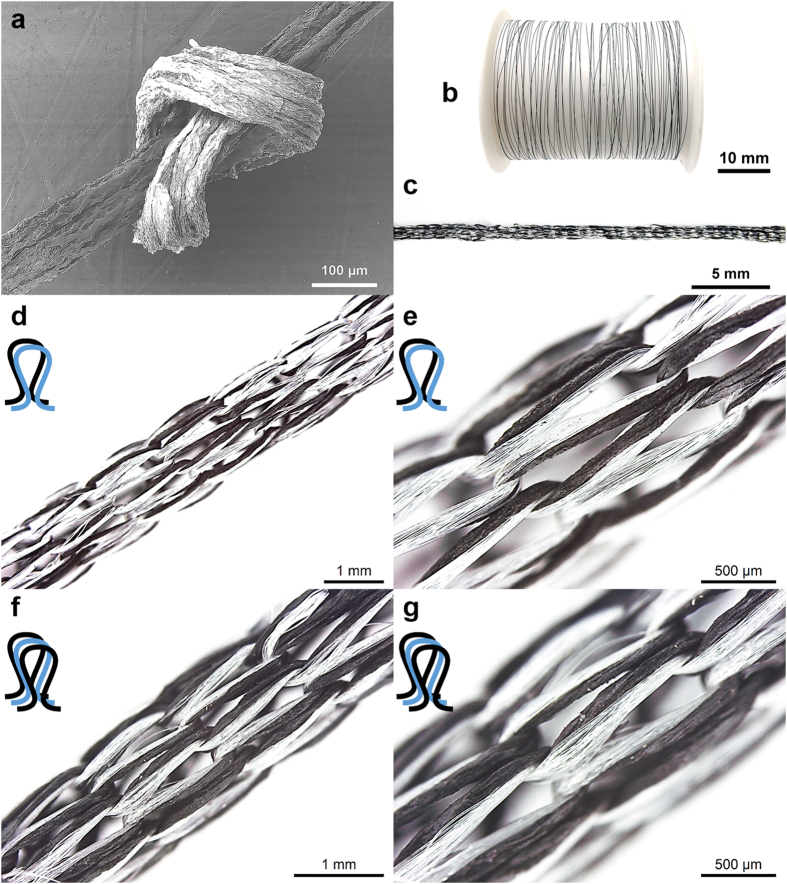
(**a**) SEM image of a GO fibre knot. Photograph of (**b**) a continuous GO fibre on a spool and (**c**) a tubular GO knitted textile. Optical microscopy images of the tubular textile structure co-knitted with a commercial nylon yarn using (**d–e**) a single and (**f–g**) double strand of GO fibre. The black loop (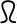) and the blue loop (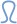) represent the GO fibre and Nylon yarn, respectively.

Some of the GO fibres produced in this work exhibited breaking strain of greater than 5% and toughness of higher than 5 MJ m^−3^. When used for knitting, these GO fibres were able to withstand the tensions applied during the movements of the fibre through the guides and the needles of the knitting machine. These fibre properties were achieved by dry-jet wet-spinning a highly concentrated spinning solution (20 mg ml^−1^) using a small nozzle diameter (30 gauge). The GO fibres produced were flexible and easily knotted (Fig. 6a). Long lengths of GO fibres were continuously produced (Fig. 6b) and co-knitted with nylon yarns into tubular knitted textiles using single and double strands of GO fibres (Fig. 6c–g). The nylon yarn used (denier 40) has a yarn diameter of ~70 μm, which is within the range of the GO fibre diameter used in knitting. Other type of yarns with dissimilar diameters than the GO fibre were also tested. Supplementary Fig. S7a shows the textile knitted from the GO fibre and a polyester yarn (100 denier) with a yarn diameter of ~100 μm. While GO fibres cannot be knitted on their own, the ability to co-knit the GO fibres with common yarns used extensively in the textile industry show great relevance for many textile-based applications. A video clip of the knitting process is also shown in the Supplementary Movie S2. GO fibres prepared using wet-spinning cannot be knitted due to the frequent breaking during knitting. Representative optical and SEM images show the detailed surface morphology of the bent portion (*i.e.* the loops) of the GO fibres in the knitted textile (Supplementary Fig. 7b-d). Upon close examination of these loops, crack formation or fibre breakage were not observed, which further supports the observed flexibility of the dry-jet wet-spun GO fibres produced in this study.

## Discussion

This work has demonstrated the feasibility of producing graphene oxide (GO) fibres that are amenable to knitting using conventional textile machinery. The knittability of the GO fibres was achieved by optimising the mechanical properties to suit the textile processing requirements in a conventional knitting machine. Detailed studies of the various fibre spinning approaches and parameters revealed the importance of jet-drawing in achieving high alignment of LCGO domains. Analysis of the fibre diameter dependence of the mechanical properties confirmed the significance of jet-drawing and gel-drawing during the fibre spinning process. It was also found that the spinning solution must have a viscoelastic gel-like behaviour to enable jet-drawing and gel-drawing. This rheological property was achieved by using high LCGO concentration and small diameter spinneret. The understanding and optimisation of these critical fibre spinning parameters have led to the production of fibres with high breaking strain and toughness, which can be successfully knitted into various textile structures using a knitting machine. These knitted textiles provide a novel platform for the next generation of functional fabrics for a broad range of applications.

## Methods

### GO dispersions

Expandable graphite flakes (3772, Asbury Graphite Mills USA) were first thermally treated at 1050 °C for 10 seconds and the resultant expanded graphite was used as the precursor for the synthesis of liquid crystalline graphene oxide (LCGO) dispersions. The full details of LCGO synthesis has been described elsewhere^4^,^17^. The GO dispersions with the desired concentration (5–50 mg mL^−1^) for fibre spinning applications were prepared by centrifugation (Eppendorf 5804) of as-synthesised GO dispersion (2.8 ± 0.1 mg mL^−1^) at 11,000 rpm (~16,000 g) for 90 min and removing the required amount of supernatant (water).

### Fibre spinning

Various configurations of wet-spinning and dry-jet wet-spinning approaches were evaluated. The wet-spinning approach^4^,^8^,^28^,^29^,^30^,^31^,^32^,^33^, was done by directly injecting the GO dispersion into a bath for the coagulation process to occur immediately. In the dry-jet wet-spinning approach, a gap of ~3 cm was introduced between the tip of the spinneret and the coagulation bath. Two configurations were used for the coagulation bath: rotary and vertical. The rotary bath configuration allows drawing of the gel-state fibre during the coagulation process by increasing the rotational speed of the bath. Minimal drawing occurs in the vertical bath configuration since the fibre is free falling. The coagulation baths were KOH (5 wt. %, Sigma-Aldrich) and CaCl_2_ (up to 10 wt. %, Scharlau) dissolved in water (MilliQ), ethanol (Chem-Supply), isopropanol (Chem-Supply), and binary mixtures of these solvents. Needles with gauges ranging from 34 to 21 (equivalent to nozzle diameters from 0.08 to 0.51 mm) were used as spinnerets and the GO dispersions were injected using a syringe pump (KD Scientific) at controlled flow rates between 1 to 10 mL h^−1^. In all cases, the GO fibres were washed with ethanol and collected onto a winder and transferred onto a spool for knitting.

### Knitting

GO fibres were co-knitted with a commercial nylon yarn (Toplon, Spin Drawn, Denier/Filament 40/24) using Harry Lucas (R1-S) circular weft knitting machine (head size 1/12”, gauge 28, 8 needles).

### Characterisation

GO sheets deposited on a silanised silicon wafer were examined under a field emission scanning electron microscope (SEM, JEOL JSM-7500FA). Silanisation was carried out by immersing the silicon substrates into a solution of 3-aminopropyltriethoxysilane (Sigma–Aldrich) in water (1:9 v/v) with an added drop of hydrochloric acid (Sigma–Aldrich) for about 30 min. GO sheets were deposited onto silanised and rinsed silicon wafers by dipping in diluted GO dispersions (50 μg mL^−1^) for about 5 seconds and then air-drying.

GO dispersions were investigated for their birefringence under a polarising optical microscope (Leica DM EP) with a 20 X objective in bright field transmission mode. Samples were prepared by transferring approximately 200 μL of the GO dispersion onto a glass slide and confining the dispersion with a cover slip and sealing the edges.

The rheological properties of the GO dispersions were studied in dynamic frequency sweep tests from 0.01 to 100 Hz at a strain magnitude of 0.01 using a rheometer (TA DHR-3) with a cone-plate geometry (cone diameter 60 mm, cone angle 2°). A strain amplitude of 0.01 was selected to avoid large deformations of the GO samples.

The morphology of the as-spun GO fibres was observed using a field emission SEM (JEOL JSM-7500FA) after sputter coating (EDWARDS Auto 306) with platinum (~5 nm). The cross-section of the GO fibres was observed from the broken ends of the fibres. Fibre diameter was measured for at least 10 points along the fibre length using an optical microscope (Leica DM EP). The mechanical properties of the GO fibres (10 samples per test) were measured using a tensile testing instrument (Shimadzu EZ-L) with a 2 N load cell and 1 N grips. Samples were prepared by attaching the fibres onto paper frames (10 mm aperture) and were then mounted on the grips after which the paper was cut in the middle. The GO fibres were stretched using a strain rate (the crosshead speed) of 1 mm min^−1^ (10% min^−1^) until failure occurred.

## Additional Information

**How to cite this article**: Seyedin, S. *et al.* Towards the Knittability of Graphene Oxide Fibres. *Sci. Rep.* **5**, 14946; doi: 10.1038/srep14946 (2015).

## Supplementary Material

Supplementary Information

Supplementary Movie S1

Supplementary Movie S2
