# Supplementary material for: Towards the Knittability of Graphene Oxide Fibres
Source: Sci Rep. 2015 Oct 13;5:14946. doi: 10.1038/srep14946 (PMC4602214; doi:10.1038/srep14946)
Supplement: Supplementary Information [file srep14946-s1.doc]

Supplementary Information

# Towards the Knittability of Graphene Oxide Fibres

Shayan Seyedin^1^, Mark S. Romano^2^, Andrew I. Minett^3^, and Joselito M. Razal^1^,*

^1^Institute for Frontier Materials, Deakin University, Geelong VIC 3216, Australia

^2^Intelligent Polymer Research Institute, University of Wollongong, Wollongong, NSW 2522, Australia

^3^Department of Chemical and Biomolecular Engineering, The University of Sydney, Sydney, NSW 2000, Australia

*joselito.razal@deakin.edu.au

**Supplementary Figure S1.** The effect of coagulation on mechanical properties of GO fibres. The coagulants (KOH or CaCl_2_) were dissolved in ethanol/water (50/50 v/v) except the last condition (ethanol only) and the GO fibres were produced by dry-jet wet-spinning method (stationary coagulation bath, GO concentration 20 mg mL^-1^, 30 gauge needle spinneret, flow rate 10 mL h^-1^).


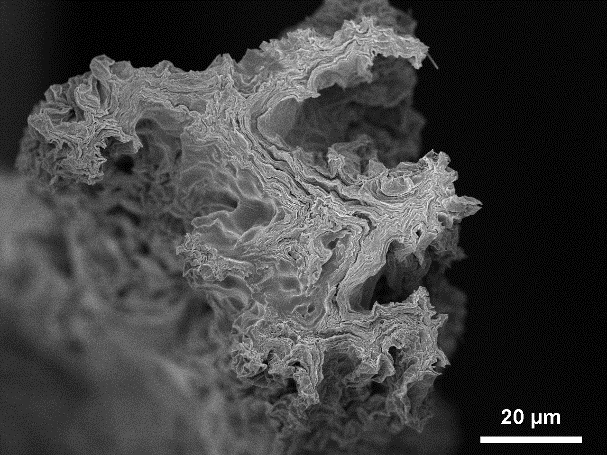

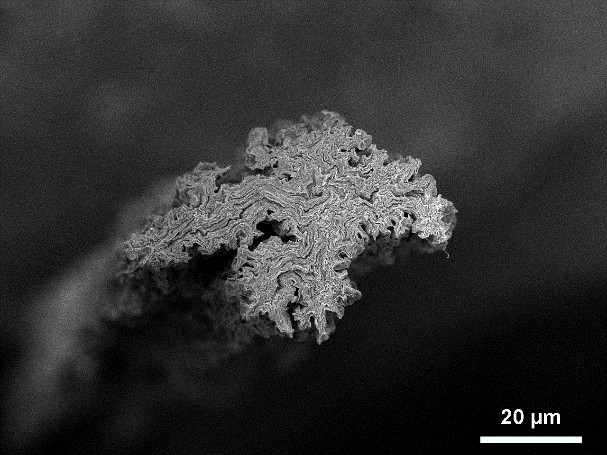

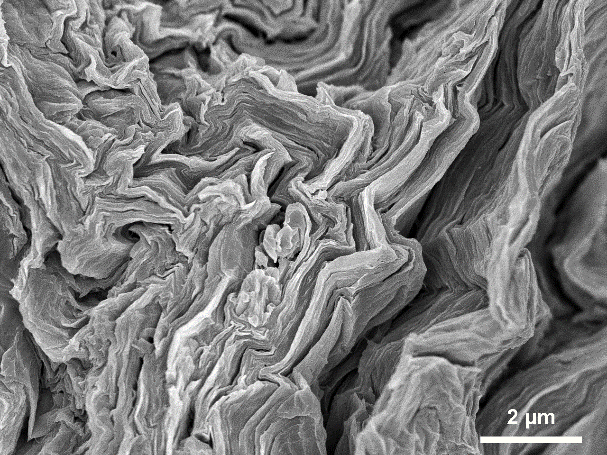

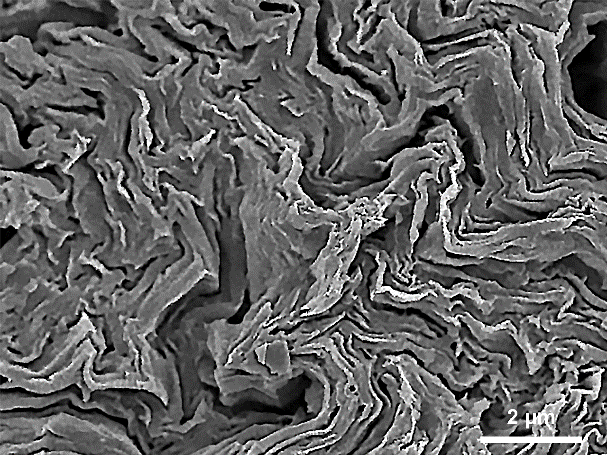

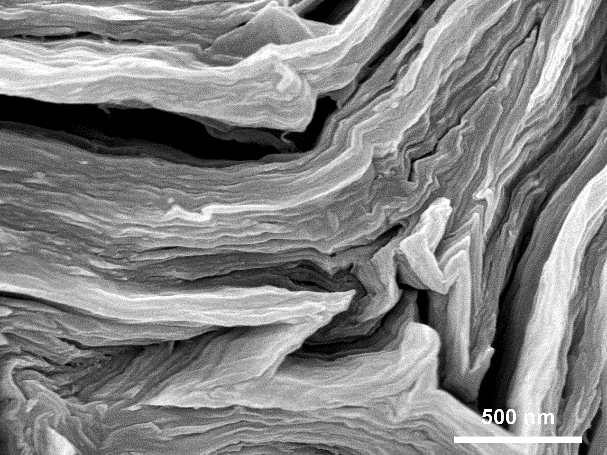

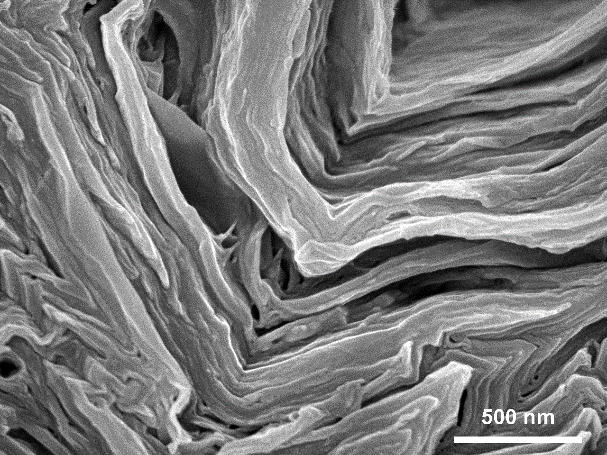


**c**

**f**

**e**

**b**

**d**

**a**

**Supplementary Figure S2.** The effect of non-solvent on cross-sectional morphology of the GO fibres. Irregular cross-section and porous morphology of the GO fibre resulted when CaCl_2_ (10 wt. %) in ethanol was used as the coagulation bath (a – c). More regular GO fibre cross-section and more packed morphology was achieved by using ethanol/water (50/50 v/v) mixture as the non-solvent (d – f). GO fibres were produced from the GO dispersion of 20 mg mL^-1^ by dry-jet wet-spinning approach with stationary coagulation bath configuration using a 30 gauge needle spinneret.

**c**

**b**

**a**

**Supplementary Figure S3.** Storage modulus (G') and loss modulus (G") as a function of frequency for different GO dispersions: a) 5 mg ml^-1^, b) 10 mg ml^-1^, c) 20 mg ml^-1^. [◼ *G'* and ⭘ *G"*]

**Supplementary Figure S4.** The effect of spinning method on mechanical properties of the GO fibres (GO concentration 20 mg mL^-1^, coagulation bath CaCl_2_ in ethanol/water (50/50 v/v), and wet-spinning with rotary and dry-jet wet-spinning with stationary coagulation bath configurations).

**Supplementary Figure S5.** Typical uniaxial tensile stress-strain curves of the GO fibres produced at different conditions. [WS: wet-spinning, DJWS: dry-jet wet-spinning, (R): rotary coagulation bath configuration]

**Supplementary Figure S6.** The effect of GO concentration and spinneret size on mechanical properties of the GO fibres (dry-jet wet-spinning with stationary coagulation bath configurations and coagulation bath CaCl_2_ in ethanol/water (50/50 v/v)).


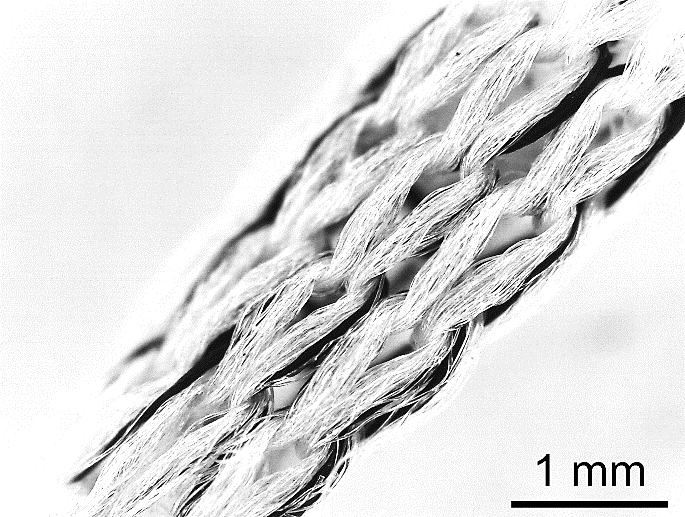

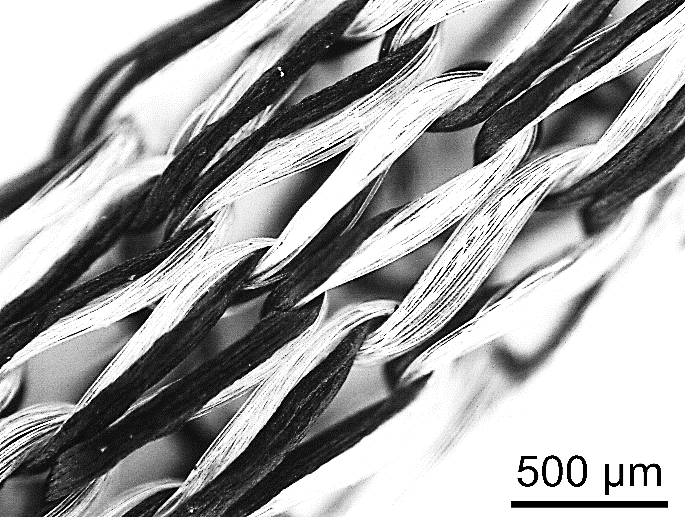


**c**

**d**

**b**

**a**

**Supplementary Figure S7.** a) optical microscopy image of the GO fibre co-knitted with a polyester yarn (100 denier). b-d) show detailed surface morphology observation of the bended GO fibres in the knitted textile under an optical microscope (b) and under SEM (c-d) at different magnifications. Arrows on (c) and (d) point to the GO fibre.

**Supplementary Movie S1.** Dry-jet wet-spinning with rotary coagulation bath configuration used for GO fibre production. Movie shows a continuous GO jet formation in air and subsequent fibre formation in the coagulation bath.

**Supplementary Movie S2.** Knitting the GO fibre with a nylon yarn using a circular weft knitting machine. Movie shows that the GO fibre can easily pass through the needles of the knitting machine without breaking.
